# Soft Regulation with Crowd Recommendation: Coordinating Self-Interested Agents in Sociotechnical Systems under Imperfect Information

**DOI:** 10.1371/journal.pone.0150343

**Published:** 2016-03-15

**Authors:** Yu Luo, Garud Iyengar, Venkat Venkatasubramanian

**Affiliations:** 1 Department of Chemical Engineering, Columbia University, New York, NY, United States of America; 2 Department of Industrial Engineering and Operations Research, Columbia University, New York, NY, United States of America; Peking University, CHINA

## Abstract

Regulating emerging industries is challenging, even controversial at times. Under-regulation can result in safety threats to plant personnel, surrounding communities, and the environment. Over-regulation may hinder innovation, progress, and economic growth. Since one typically has limited understanding of, and experience with, the novel technology in practice, it is difficult to accomplish a properly *balanced* regulation. In this work, we propose a control and coordination policy called *soft regulation* that attempts to strike the right balance and create a collective *learning* environment. In soft regulation mechanism, individual agents can accept, reject, or partially accept the regulator’s recommendation. This non-intrusive coordination does not interrupt normal operations. The extent to which an agent accepts the recommendation is mediated by a confidence level (from 0 to 100%). Among all possible recommendation methods, we investigate two in particular: the best recommendation wherein the regulator is completely informed and the crowd recommendation wherein the regulator collects the crowd’s average and recommends that value. We show by analysis and simulations that soft regulation with crowd recommendation performs well. It converges to optimum, and is as good as the best recommendation for a wide range of confidence levels. This work sheds a new theoretical perspective on the concept of the wisdom of crowds.

## Introduction

Regulating emerging technologies is challenging, and often controversial; requiring a careful trade-off between stability, security, performance, and cost in an *uncertain* environment. Recent examples of emerging technologies include hydraulic fracturing, carbon sequestration, deep sea mining, geoengineering, and personalized medicine. Hydraulic fracturing, for example, has grown to be a transforming force in the petrochemical industries in recent years with its proponents and opponents debating passionately about its benefits and costs to the society with the attendant regulatory challenges [1, 2].

The regulator’s (or central planner’s) dilemma with regard to emerging technologies is to strike the appropriate *balance* in regulation. Under-regulation can result in damage to plant personnel, surrounding communities, and the environment. Over-regulation, on the other hand, can hamper economic growth and security. When a technology is new, the inherent risks and benefits are not immediately obvious and only become clear over time, making it harder for the regulatory agency to strike the correct balance in the early stages. This uncertainty necessitates a framework that allows for a very close collaboration between the regulatory agency and the regulated entities that have direct access to field performance, and hence have direct knowledge of what worked and what did not.

In a typical regulatory environment involving conventional technologies, regulators issue mandates that have to be followed by the regulated agents. The agents face fines and other punitive consequences for non-compliance. In this paper, we will call this approach *hard regulation*. We argue that hard regulation is not very effective for regulating emerging technologies. Hard regulation also hinders innovation [3]. The regulation of the Internet illustrates these issues very well. Laws like Digital Millennium Copyright Act (DMCA) and Stop Online Piracy Act (SOPA) have been criticized [4–8], as they arguably “reduce freedom of expression and undermine the dynamic, innovative global Internet.” In addition, while attempting to protect intellectual property, these laws hurt computer security by inhibiting research on security related issues [9]. During the period when a new technology is still maturing, the regulator is just as unsure as the regulated agents about the risk-benefit trade-off, and therefore, hard regulation, through its unintended consequences, could potentially do more harm than good. Instead of issuing potentially misdirected mandates, the regulator and the agents should jointly participate in learning about the emerging technology and its payoff structure. The focus of this work is on how to achieve this elusive goal through an intellectual framework that facilitates both *control* and *learning* in sociotechnical systems.

Control and learning are essential elements in managing risk and regulating behavior in sociotechnical systems (see Table 1). In a purely technical setting, i.e., when all the elements of the systems are machines, the common practice to maintain an efficient and stable system is to use *hard* control where the entities follow strictly specified policies. Process control, robotics, etc., are all examples of hard regulation or hard control. When there is no reliable model or a desirable set point available, one needs to simultaneously learn and control the system dynamics. We call techniques, such as machine learning, stochastic approximation, etc., *hard* learning techniques since they also require the entities to follow strict instructions.

**Table 1:** Control and learning in sociotechnical systems.

	Control	Learning
**Hard**	feedback control, model predictive control, hard regulation, robot formation, laws, etc.	machine learning, stochastic approximation, Kalman filter, evolutionary dynamics, etc.
**Soft**	persuasion, soft paternalism, peer pressure, social engineering, mechanism design, etc.	social sensing, social learning, pervasive mobile computing, etc.

However, in a sociotechnical system with active human participants, hard control, or strict mandates, may not always be appropriate. Mandates can potentially do more harm than good as we argued earlier. A more appropriate course of action would be to offer options to agents that are adopted only when they are incentive compatible. We call such approaches *soft* control [10, 11]. Examples of this approach include the soft paternalism approach for modifying social behavior [12] wherein carefully designed options “nudge” people to make better decisions [13]; or policy teaching [11] wherein the regulator allocates rewards in such a way that the induced action of agents maximizes the regulator’s value. Other examples include efforts by utility companies to induce consumers to minimize power wastage by reporting average consumption [14]; and health tracking devices, e.g., *Fitbit*, *Jawbone Up*, *Nike FuelBand*, etc., that all incorporate social nudging to motivate physical activities. The soft control policy using peer pressure is shown to promote cooperation in these and other settings, both in theory and in practice [15–18].

As in the case with hard control, soft control can be used only when there is a reliable model and a well-defined set point. Soft paternalism and similar social mechanisms are effective because we understand saving energy and staying physically active are the right things to do. What if we do not know what is best for the agents? Soft learning is a class of learning mechanisms that appropriately incentivize agents in a social network to aggregate important information. Examples of soft learning include social sensing and social learning [18–20] in the context of real-time traffic information and online reviews (such as Yelp).

In this work, we propose *soft regulation* as a new regulatory paradigm that combines features of soft control and soft learning. The regulator aggregates key system-level statistics in a privacy preserving [21] manner (individuals do not need to explicitly disclose their states) and shares these statistics with all agents. The agents have the flexibility to accept, reject, or partially accept the recommendations from the regulator based on their own self interests. The recommendations are simply “nudges” [13]. The mechanism does not interrupt the regulated entities who have direct access to field performance. It creates a collective learning environment for both the regulator and the agents. This partial acceptance (or confidence level) of recommendation is a crucial feature of soft regulation. It critically determines the effectiveness of the mechanism. Soft regulation seeks a balance between over- and under-regulation: Agents have the freedom to rely on both individual exploration and social learning.

We expect soft regulation to be effective when the system has the following features:

1. *Imperfect information*: The action-utility payoff structure is poorly understood, i.e., the data are *noisy* and the models are absent or *incomplete*. Each individual may only possess partial information about the unknown process. Agents rely on inaccurate measurements, approximations, or subjective evaluations to optimize. Later we will discuss how noise, paradoxically, is necessary for soft regulation to add value.
2. *Weak interaction*: The agents can optimize their own actions without taking into consideration the response of other agents, i.e., each’s utility or payoff is only a function of the agent’s own state, and the optimal set point is identical among agents. A good example of such a setting is the initial stages of a new technology; the resources being exploited are abundant and the profits of the agents are not limited by competition but by their ability to exploit the resource effectively. Although the reward an agent receives while operating at a set point may vary, the set point itself, however, is likely to be identical or at least restricted to a narrow range. The discovered set points (by soft regulation or traditional methods) will later become the industry standards when the technology matures. Another example of setting with weak or no interaction is when humans improve their own health conditions by changing habits, medications, or even environments. The interaction among agents is usually minimal. Although each has his/her own unique physiological configurations, grouped by characteristics such as age, gender, profession, etc., they are likely to exhibit common optimal set points within groups.
3. *Bounded rationality*: Agents are autonomous and self-interested, and they *always* move in a direction that locally improves utility, subject to available information.

Soft regulation creates a feedback system where agents have the freedom to choose to accept this feedback. Feedback has long been recognized as an essential feature of complex adaptive systems where causes and effects are intertwined. There have been several attempts over the years to understand the dynamics of social systems in terms of feedback control (see, e.g., [22–25]). While such contributions are useful advances, much of this work, however, is conceptual and qualitative. In contrast, soft regulation is a practical and quantitative methodology—self-interested agents use the feedback from past outcomes to determine future actions, and the regulator provides all the agents a feedback that aggregates system-level information. One can extend this to include group, organization, even societal-level feedback loops. In this study, we focus on the bottom two levels of such a feedback control hierarchy.

There are several ways to generate feedbacks for soft regulation. We focus on two methodologies in this paper: the best recommendation and the crowd recommendation. As the name suggests, best recommendation corresponds to the case where the regulator has full information and computes the feedback by solving a *centralized* optimal control problem. The crowd recommendation on the other hand, is simply the average of the agents’ actions. We show that, despite its simplicity, crowd recommendation is as good as the best recommendation for a wide range of confidence levels. This is unsurprising. The collective wisdom of groups has been acknowledged in the literature; see, e.g., the Condorcet’s jury theorem [26] and popular bestsellers such as *The Wisdom of Crowds* [27] and *Wiser: Getting Beyond Groupthink to Make Groups Smarter* [28]. In this work, we propose a control-theoretic and mathematical framework that goes beyond one-time predictions and investigates the effectiveness of the wisdom of crowds for optimizing a process using continuously refined information.

The scope of this paper is twofold. We first introduce the concept of soft regulation where agents have the freedom to partially accept the feedback from the regulator. We then investigate a unique and practical way of implementing soft regulation—regulator issuing crowd recommendation. In the remaining paper, we formally define the problem and show by analysis and simulation that soft regulation with crowd recommendation indeed performs well.

## Background

In this paper, we analyze a stylized model of soft regulation that preserves the essential features discussed in the introduction. The system consists of one regulator and *n* agents. Agent *i* wants to select an action *x*_*i*_ that maximizes the value of the real-valued and strongly concave utility function *f*_*i*_(*x*_*i*_) over a convex compact set X⊆R. We assume that although the individual utility functions *f*_*i*_ are different for each agent, the optimal set point $\theta^{*}=\arg\max_{x\in X}f_{i}\left(x\right)$ is identical across agents.

We assume that the utility function *f*_*i*_(*x*_*i*_) is not explicitly known, nor is it deterministic; agents cannot solve the optimization problem explicitly. In theory, by averaging out the noise, one can obtain a more accurate mapping of the utility function. However, in our setting, each sample corresponds to actual utility each agent receives; therefore, they might not have the incentive to oversample at the location where the utility is low. The agents update individual actions using the following dynamics:

$$\begin{array}{c} {\bar{x}}_{i}=g_{i}\left(x_{i}\right) \end{array}$$

where *g*_*i*_ denotes the optimization algorithm used by the *i*-th agent. In practice, *g*_*i*_ can be any function that maps an old action *x*_*i*_ to a new action ${\bar{x}}_{i}$. In order to converge to the optimal θ*, the function must satisfy regularity conditions. More specifically, *g*_*i*_ should converge to a unique fixed point regardless of the initial value of *x*_*i*_. Although the conclusions of this paper are applicable to a wide range of optimization methods, here we focus on a commonly used algorithm named Kiefer-Wolfowitz stochastic gradient method [29] where

$$\begin{array}{c} g_{i}\left(x_{i}\right)=x_{i}+\frac{a(t)}{c(t)}\cdot \left(f_{i}\left(x_{i}+c\left(t\right)\right)-f_{i}\left(x_{i}-c\left(t\right)\right)\right). \end{array}$$ (1)

At time *t*, the *i*-th agent samples the payoff twice at the vicinity of its current state *x*_*i*_, which is only known to the agent. The parameters *a*(*t*) and *c*(*t*) are known and predefined. The agent then computes the next step according to Eq (1). This algorithm is guaranteed to converge in probability when

$$\begin{array}{c} E{\left[f_{i}-E\left[f_{i}\right]\right]}^{2}<\infty ,\;\lim_{t\to \infty }c\left(t\right)=0,\;\sum_{t=1}^{\infty }a\left(t\right)=\infty ,\;\sum_{t=1}^{\infty }\frac{a{\left(t\right)}^{2}}{c{\left(t\right)}^{2}}<\infty . \end{array}$$

We call a setting where an agent updates its action based on its own measurement the *open loop* scenario (or asocial learning as in [20]).

In the soft regulation setting the regulator computes a feedback recommendation *u*. The agents then combine *u* with ${\bar{x}}_{i}=g_{i}\left(x_{i}\right)$ to compute a new action $x_{i}^{+}$ in the following manner:

$$\begin{array}{c} x_{i}^{+}=h_{i}\left(x_{i}\right)\equiv \left(1-\beta_{i}\right)g_{i}\left(x_{i}\right)+\beta_{i}u=\left(1-\beta_{i}\right){\bar{x}}_{i}+\beta_{i}u \end{array}$$

where *β*_*i*_ ∈ [0, 1] or [0, 100%] is a measure of the *confidence* that the *i*-th agent puts on the feedback, and is therefore, called the confidence level. Note that we use $x_{i}^{+}$, *x*_*i*, *t*+1_, and *x*_*i*_(*t* + 1) notations interchangeably. Subscript *t* is omitted in most situations. The confidence level *β* plays an important role in the resulting dynamics. Action changes are relatively independent of recommendation for agents with small *β* (the *explorers*), and action remains in the vicinity of *u* for agents with large *β* (the *followers*).

Note that soft regulation is not an example of direct social learning as described in [20]: There is no “best agent” to follow because the payoffs are private information and noisy. That said, explorers do resemble the asocial innovators and the followers resemble the copying agents in the social learning setting [20]. The confidence level of an agent may be indirectly related to peer pressure [15–18]: The followers experience a higher peer pressure than the explorers, and therefore, set a higher value of *β*. Also note that *h*_*i*_(*x*_*i*_) can be re-written as follows:

$$\begin{array}{c} h_{i}\left(x_{i}\right)=x_{i}+\left(1-\beta_{i}\right)\left({\bar{x}}_{i}-x_{i}\right)+\beta_{i}\left(u-x_{i}\right). \end{array}$$

The soft regulation feedback function resembles the feedback seen in bird flocks and swarm intelligence [30].

When the regulator is fully informed about the functions *f*_*i*_, *g*_*i*_, and *β*_*i*_, the optimal feedback u* can be computed explicitly by solving the following centralized optimal control problem that maximizes social welfare (sum of utilities) over the projected trajectory:

$$\begin{array}{cc} \text{maximize} & \sum_{t}w\left(t\right)\sum_{i}f_{i}\left(x_{i}\left(t\right)\right)\\ \text{subject to} & x_{i}\left(t+1\right)=\left(1-\beta_{i}\right)g_{i}\left(x_{i}\left(t\right)\right)+\beta_{i}u\left(t\right) \end{array}$$

where the time varying weight *w*(*t*) can favor either the present or future. We call the solution u* to this problem the best recommendation.

Since the function *f*_*i*_, *g*_*i*_, and the parameter *β*_*i*_ are only privately known to the agents, in practice, it is unlikely that the regulator knows the functions and the parameters. Following [14, 27, 28], we assume that the regulator reports the average, i.e., $u=\frac{1}{n}\sum_{i}x_{i}$. We call this recommendation the crowd recommendation. Note that using privacy preserving computations [21], the regulator can compute the crowd recommendation without ever learning any individual input *x*_*i*_. In the rest of this paper, we demonstrate that the crowd recommendation ensures the convergence to the optimal set point; moreover, it is as good as the best recommendation for a wide range of confidence levels.

## Optimality, robustness, and efficiency of soft regulation

Optimality, robustness, and efficiency are three important characteristics of a mechanism. We define an *optimal* process as one that converges to the maximum utility eventually, a *robust* process as one that the equilibrium can be restored when subjected to disturbances, and an *efficient* process as one that converges quickly to the optimum.

We first show that soft regulation with crowd recommendation is *optimal* and *robust* when subjected to bounded noises if the individual confidence level *β*_*i*_ satisfies 0 ≤ *β*_*i*_ < 100%. We describe the essential steps of the proof in this section. Interested readers can find detailed derivations in S1 Appendix.

The nominal (noise free) optimality of soft regulation can be proved using contraction. Recall our assumption that an agent always adopts an optimal algorithm *g*_*i*_ to find θ* that maximizes utility *f*_*i*_. In other words, the map defined by *g*_*i*_ converges to θ* regardless of the initial condition, i.e., *g*_*i*_ is a contraction and θ* is its unique fixed point. Consequently, there exists a constant *c* < 1 such that |*g*_*i*_(*x*) − *g*_*i*_(*y*)| ≤ *c*|*x* − *y*|. If *g*_*i*_ is differentiable; then we have $|g_{i}^{\prime }\left(x\right)|<1$ for all x∈X. The soft regulation process is *h*_*i*_ = (1 − *β*_*i*_)*g*_*i*_ + *βu* where $u=\frac{1}{n}\sum_{i}x_{i}$ is the crowd recommendation. We define column vector **x** = [*x*_1_, …, *x*_*n*_]^*T*^ as the system state and **x**^+^ = *H*(**x**) as the system-wide soft regulation map. Whether a map contracts is determined by the eigenvalues of its Jacobian matrix. We can show that the largest absolute eigenvalue of the Jacobian is strictly less than 1 whenever 0 ≤ *β*_*i*_ < 100%. Thus, soft regulation with crowd recommendation is also optimal. Note that this proof is applicable to a completely heterogeneous system where both the optimization algorithm *g*_*i*_ and the confidence level *β*_*i*_ are different among individuals. See S1 Appendix for detailed derivations.

According to stability theory [31], the optimal state x* = [θ*, …, θ*]^*T*^ is robust to bounded disturbances if and only if there exists a *continuous* Lyapunov function for the soft regulation process **x**^+^ = *H*(**x**). We show that *V*(**x**) = ‖**x** − x*‖_1_, i.e., the Manhattan distance or *ℓ*_1_-norm between the current state **x** and the optimal state x*, is a Lyapunov function for the process. The contraction result is used to show that such function *V*(**x**) is acceptable and the optimal state is robust. This completes the robustness proof.

We use current mean squared error (MSE) to indicate the efficiency of soft regulation, i.e.,

$$\begin{array}{c} \text{MSE}=\frac{1}{n}{∥x-x^{*}∥}_{2}^{2}. \end{array}$$

Here we are interested in how *β* affects the MSE. We simplify the analysis by setting *β*_*i*_ ≡ *β*. From mean value theorem for vector-valued function, we have

$$\begin{array}{c} E\left[{\text{MSE}}^{+}\right]\leq m^{2}\text{MSE}+{\left(1-\beta \right)}^{2}\sigma_{\omega }^{2} \end{array}$$

where $m=\left(1-\beta \right)\max_{1\leq i\leq n,x\in X}\left|g_{i}^{\prime }\left(x\right)\right|+\beta <1$ is the upper bound of contraction and *σ*_*ω*_ < ∞ is the standard deviation of the zero-mean noise imposed on *g*_*i*_. The MSE evolution exhibits first order dynamics: Increasing *β* (or increasing *m*) decreases the steady state expected MSE; however, a larger *β* leads to a larger *m*, and therefore, a weaker contraction and slower convergence. In a noisy system where *σ*_*ω*_ is large, it is advisable for the agents to rely more on the recommendation using a larger *β*. There is a trade-off between accuracy and speed. This further implies that at a finite time *t*, there should exist an optimal β* between 0 and 100% such that the MSE is the smallest.

## Simulation of finite-time dynamics

For practical applications, it is more important to understand the transient or *finite-time* dynamics of soft regulation, and more specifically, the role of confidence level in setting the transient performance. We are able to illustrate with a few additional assumptions about the system.

Recall that, we assume the underlying action-utility payoff function *f*_*i*_(*x*_*i*_) to be strongly concave. In this section, our analysis will be focused on the *simplest* concave function, namely an identical and quadratic utility function *f*_*i*_(*x*_*i*_) = *f*(*x*_*i*_) = −*k*(*x*_*i*_ − θ*)^2^ + *ω*. Identical agents are helpful in identifying the effect of confidence level. The results can be readily extended to heterogeneous systems. In order to study the convergence behavior, one can without loss of generality, assume that θ* = 0. This particular choice for *f* is motivated by the fact that any strongly concave function can be approximated by a quadratic function near its optimum. The noise is $\omega ∼N(0,\sigma_{\omega })$. Agents only observe the noisy function values—the underlying structure is not known to the agents.

We define the optimization *efficiency* as the percent reduction in MSE:

$$\begin{array}{c} \eta_{t}\equiv \frac{{\text{MSE}}_{t_{0}}-{\text{MSE}}_{t}}{{\text{MSE}}_{t_{0}}}\times 100. \end{array}$$

The efficiency is 100 when the system reaches optimum. We simulate the agent dynamics in NetLogo. The agent set is randomized by a fixed random seed in the program to ensure consistency. For each set of parameters, we run the simulation five times and take the average. The observed deviation was insignificant; therefore we omitted error bars. The model parameter values are listed in Table 2. The noise $\omega ∼N(0,\sigma_{\omega })$ has the same variance as another random variable $\epsilon ∼\text{unif}(-\sqrt{3}\sigma_{\omega },\sqrt{3}\sigma_{\omega })$. We chose *σ*_*ω*_ to be $200/\sqrt{3}$ so that it is computationally equivalent to a uniform ±200 noise. The parameters do not represent practical meanings. The particular values are chosen such that the results are easily identifiable.

**Table 2:** Model parameters.

*n*	*σ*_*ω*_	θ*	*k*	*a*(*t*)	*c*(*t*)
1000	$200/\sqrt{3}$	0	100	1/*t*	1/(*t* + 200)^1/3^

We first run the simulation for soft regulation with best recommendation. Given the quadratic utility, Kiefer-Wolfowitz algorithm, and system-wide confidence level, the regulator can easily compute best recommendation by solving the optimal control problem introduced earlier. One can obtain the nominal (without noise) system dynamics to be

$$\begin{array}{c} x(t+1)=\left(1-\beta \right)\left(1-4ka_{t}\right)x\left(t\right)+\beta 1u\left(t\right). \end{array}$$

Since the stage cost does not penalize input *u*, the optimal u* at stage *t* can be solved as follows

$$\begin{array}{c} u^{*}\left(t\right)=-\left(\frac{\left(1-\beta \right)\left(1-4ka_{t}\right)}{\beta }\right)\cdot \frac{1}{n}\sum_{i}x_{i}\left(t\right). \end{array}$$

**Fig 1:**
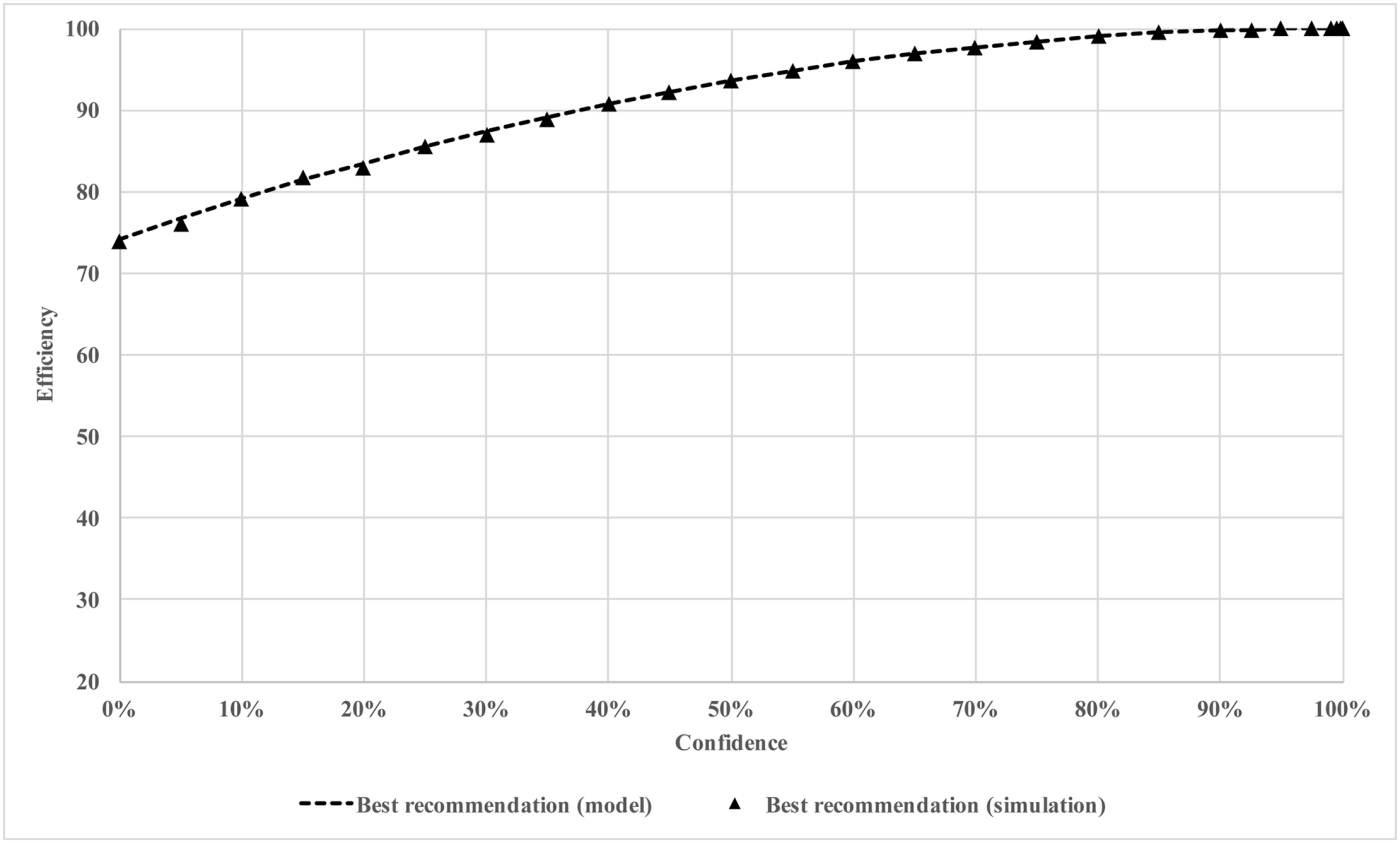
Efficiency of soft regulation with best recommendation.

In Fig 1, we plot the efficiency after 200 iterations against different confidence levels. We observe the efficiency increases monotonically as the confidence level increases. This result is not surprising. As the confidence level increases, the regulator has a stronger influence on the agents, therefore, exerting a more efficient control. Even though for each confidence level, the regulator issues the best recommendation, the recommendation is only effective when the agents choose to listen.

**Fig 2:**
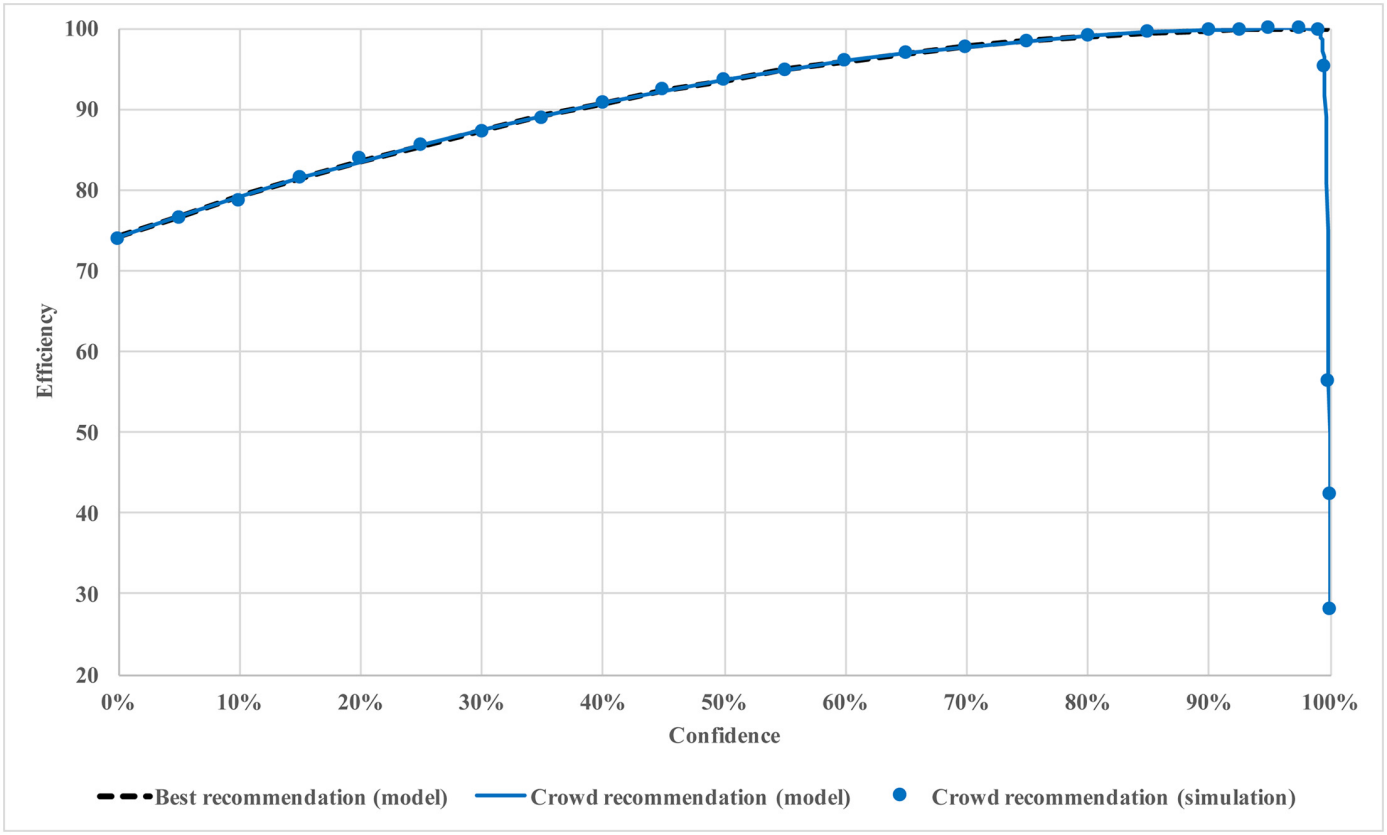
Efficiency of soft regulation with crowd recommendation.

**Fig 3:**
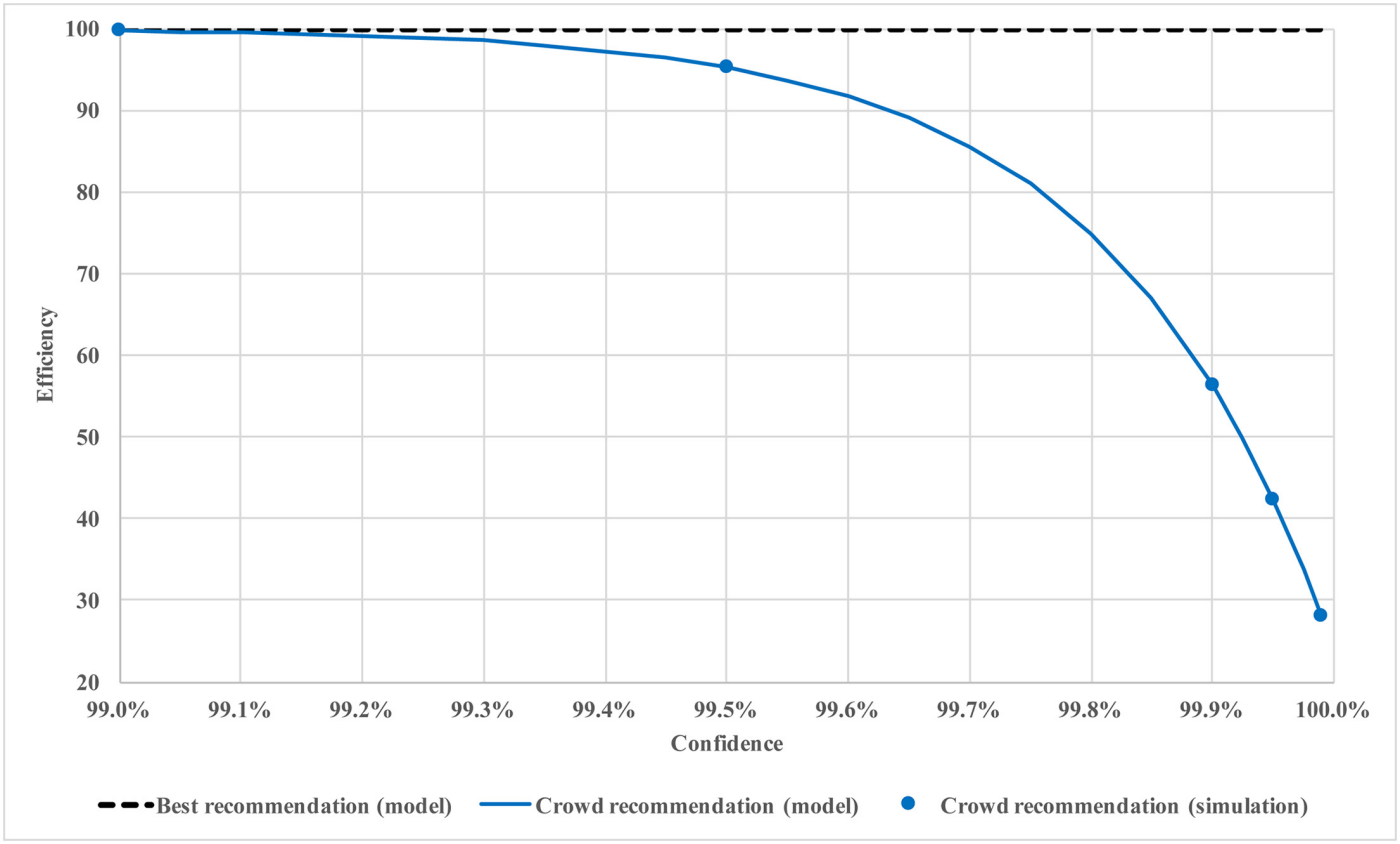
Efficiency of soft regulation with crowd recommendation (large confidence levels).

In Figs 2 and 3, we plot the efficiency against confidence level for soft regulation with crowd recommendation. The results from Fig 1 are also included as a reference. It is remarkable that soft regulation with crowd recommendation is as good as the one with best recommendation for a wide range of confidence levels (from 0 to 99%). The real advantage of best recommendation only appears when the confidence level is close to 100%. However, to achieve this best recommendation or even hard regulation, the regulator needs information about utility function, optimization algorithm, and the confidence level. This practice, despite being efficient under the setting of complete information, is costly, impractical, and error prone in practical settings. Especially for hard regulation, additional cost of enforcement needs to be considered.

The results in Figs 2 and 3 confirm our previous insights that the confidence level should be set to a large value but not too close to 100%. The open loop system only reaches about 70% optimum. The system performance is more than 90% optimal when the confidence level is 50% (i.e., the agent takes an average between its own optimization result and the recommendation). We also see a sharp decline in performance when confidence level is too close to 100%. Beyond this “cliff,” the agents explore very little and essentially stay where they are.

**Fig 4:**
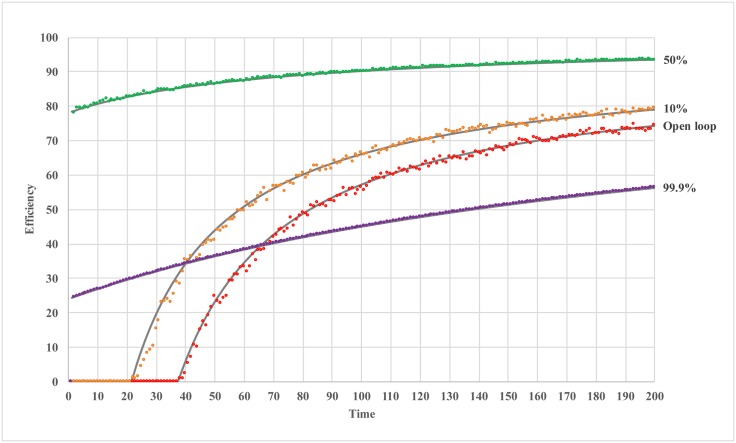
Efficiency of soft regulation with crowd recommendation over time.

In Fig 4, we plot the time progressions of efficiency for different confidence levels. When confidence level is low (*β* = 0 or 10%), the MSE increases (efficiency declines) before converging. This is caused by large initial step sizes. As confidence level increases, the system begins convergence earlier. As the confidence level further increases, the system shifts from the regime dominated by exploration to the one dominated by conformity, and the recommendation does not have enough time to converge to optimum before agents start conforming.

In order to better understand the connections between the confidence level and the performance that we hypothesized in the previous section and observed in the simulation results, we now attempt to compute a *closed-form* expression for the system state. Recall that *x*_*i*_ denotes the current state of the *i*-th agent. The updated state $x_{i}^{+}$ implied by the gradient-based update scheme in Eq (1) is given by

$$\begin{array}{c} x_{i}^{+}=\left(1-\beta \right)\left(1-4ka_{t}\right)x_{i}+\beta u+a_{t}\cdot \frac{1-\beta }{c_{t}}\cdot {\hat{\omega }}_{i} \end{array}$$ (2)

where ${\hat{\omega }}_{i}∼N\left(0,\sqrt{2}\sigma_{\omega }\right)$ is the effective noise resulting from computing the discrete approximation to the gradient in Eq (1). Recall that the crowd recommendation is *u* = ∑_*i*_ *x*_*i*_/*n*. Thus, the updated *u*^+^ of the recommendation is given by

$$\begin{array}{c} u^{+}=\left[1-4ka_{t}\left(1-\beta \right)\right]u+a_{t}\cdot \frac{1-\beta }{c_{t}}\cdot \frac{1}{n}\sum_{i}{\hat{\omega }}_{i}. \end{array}$$

When *n* ≫ 1, the expected *u*(*t*) can be treated as a deterministic variable. When *β* is small, *u*(*t*) quickly converges to θ*. Otherwise, E[u(t)] can be approximated as follows:

$$\begin{array}{c} E\left[u\left(t\right)\right]\approx u\left(t_{0}\right)\exp\left[-4k\left(1-\beta \right)\int_{t_{0}}^{t}a_{\tau }d\tau \right]. \end{array}$$ (3)

For the wisdom of crowds, this implies that a group is smart only when the population is large (*n* ≫ 1) and agents are not strongly conforming (*β* ≪ 100%). Surowiecki’s book [27] shares the same insights. Unlike the averaging method in the book, soft regulation is a continuous feedback process. Even though the open loop (*β* = 0) system has the fastest converging recommendation, agents cannot make use of it unless they at least partially accept (*β* > 0). This paradox suggests some trade-off and balancing between consensus and efficiency.

In addition, as *k* increases, *u*(*t*) approaches θ* faster, i.e., a more sensitive utility function implies a more reliable recommendation. Unless an agent can estimate the curvature (∼*k* for a quadratic function) of the payoff accurately, it is safer to rely on the recommendation when curvature is larger.

From Eqs (2) to (3) it follows that

$$\begin{array}{c} E[\text{MSE}(t+1)]\approx \frac{2a_{t}^{2}{\left(1-\beta \right)}^{2}\sigma_{\omega }^{2}/c_{t}^{2}+\beta \left[2\left(1-\beta \right)\left(1-4ka_{t}\right)+1\right]u{\left(t\right)}^{2}}{1-{\left(1-\beta \right)}^{2}{\left(1-4ka_{t}\right)}^{2}}. \end{array}$$ (4)

This approximation agrees closely with the simulation (see lines in all simulation result figures). Interested readers can find detailed derivations in S1 Appendix.

From Eq (4), it follows that MSE converges to 0 (soft regulation is optimal). Meanwhile, when confidence level is low, i.e., *β* ≈ 0 (explorers), the dependence on *u*(*t*) vanishes very quickly, and Eq (4) can be simplified as follows:

$$\begin{array}{c} E\left[\text{MSE}\left(t+1\right)\right]\approx \frac{2a_{t}^{2}\sigma_{\omega }^{2}/c_{t}^{2}}{1/{\left(1-\beta \right)}^{2}-{\left(1-4ka_{t}\right)}^{2}} \end{array}$$

and MSE monotonically *decreases* as *β* increases. On the other hand, when confidence level is high, i.e., *β* ≈ 100% (followers), the *u*^2^(*t*) term dominates $\sigma_{\omega }^{2}$, one can simplify Eq (4) to

$$\begin{array}{c} E\left[\text{MSE}\left(t+1\right)\right]\approx \beta {\left\{u\left(t_{0}\right)\exp\left[-4k\left(1-\beta \right)\int_{t_{0}}^{t}a_{\tau }d\tau \right]\right\}}^{2} \end{array}$$

and MSE monotonically *increases* as *β* increases. This estimation agrees well with our previous hypotheses and simulation results (see Figs 2 to 4).

An interesting fact arises from this approximation, i.e., imperfect information is necessary for soft regulation to add value. If the system has very low noise or noise-free, the $\sigma_{\omega }^{2}$ term will be dominated by *u*^2^(*t*), and an increase in *β* hurts performance. That is to say, *for a deterministic process, soft regulation with crowd recommendation may not be a good mechanism for agents to adopt*.

In practice, each individual may have a distinct confidence level and personal traits. Modeling such rich details as well as formulating related best recommendation is beyond the scope of this work. Nevertheless, for purposes of illustration, we propose the following adaptive confidence mechanism:

$$\begin{array}{c} \beta \left(x_{i},u\right)=e^{-b{\left(x_{i}-u\right)}^{2}}\;\left(b>0\right). \end{array}$$

**Fig 5:**
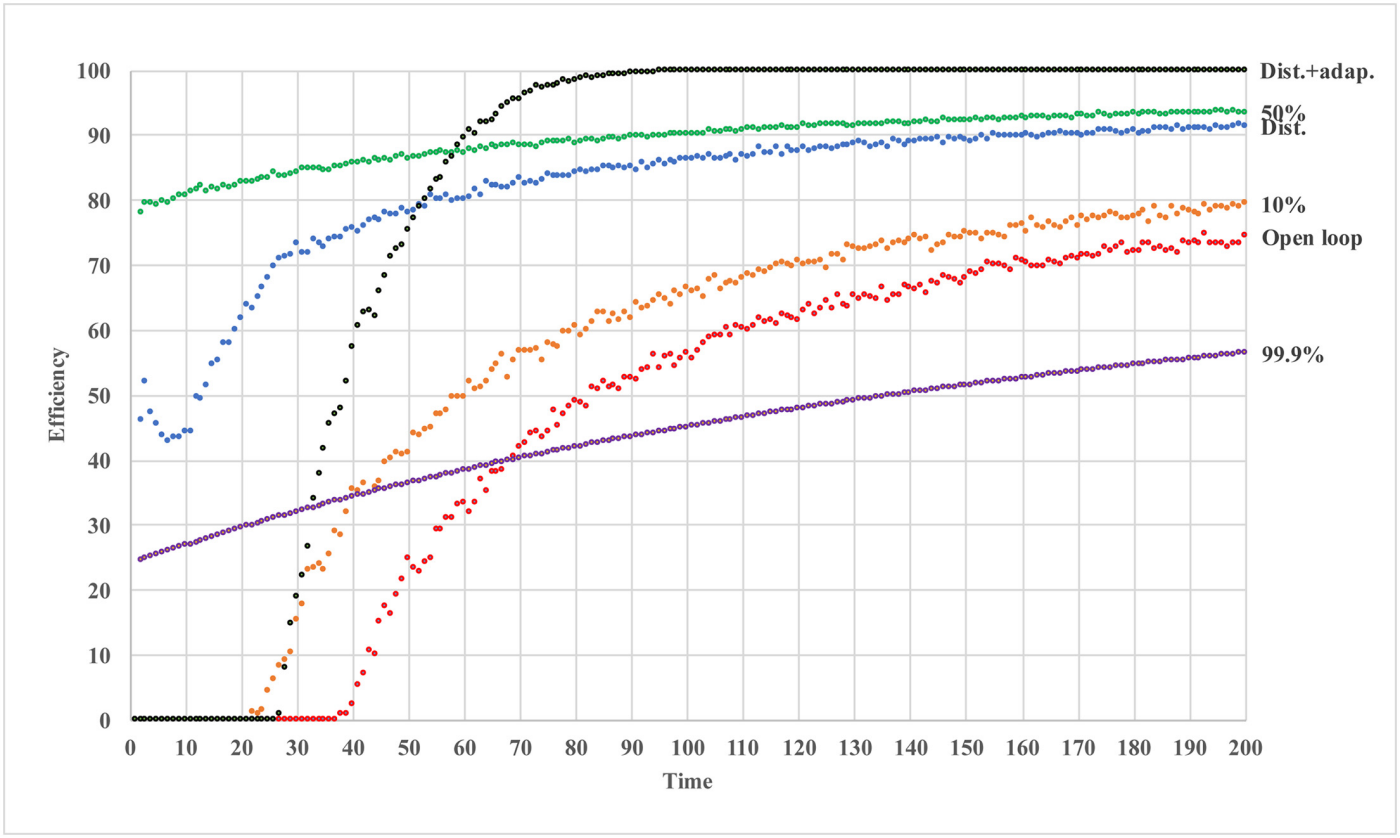
Efficiency of soft regulation with crowd recommendation over time (distributed agents).

The rationale for this update scheme is as follows. When an agent’s action is far away from the recommendation, the agent is fairly skeptical. Suppose, by incorporating the recommendation, the agent’s action moves further away from the recommendation, the agent would rely even less on the regulator. However, when the action comes closer to the recommendation, agent is likely to be more confident about the regulator, and incorporate the recommendation in future updates. One flaw in this adaptive mechanism is that if everyone performs the same action in the beginning, this results in an identical confidence level *β* = 100% for all agents, and the system will not move at all. This situation might be remedied with an occasional, small perturbation. In Fig 5, we plot two new simulation results, i.e., 1) agents have uniformly distributed (*Dist*.) confidence levels, and 2) agents have uniformly distributed initial confidence levels and the confidence is adaptive (*Dist.+adap*.) according to the update scheme above. We also include previous results with fixed and identical confidence level to the graph. We observe a fairly good performance.

## Conclusion

We propose a soft regulation framework for coordinating multi-agent systems. In this setting, the regulator’s role is to help agents learn, understand, and optimize an unknown process without interrupting normal operations. The essence of this mechanism is to take guided decisions by updating actions using the map $x_{i}^{+}=\left(1-\beta_{i}\right){\bar{x}}_{i}+\beta_{i}u$, where ${\bar{x}}_{i}$ is the *i*-th agent’s own optimized decision and *u* is the regulator’s crowd recommendation. Self-interested agents have the freedom to choose to partially accept the regulator’s recommendation. Soft regulation provides a more balanced coordination: Unlike hard regulation, it does not force the agents; this creates a collective learning environment and avoids possibly erroneous mandates. On the other hand, a soft regulatory system is not under-regulated or uncoupled. The exploration of some agents benefits others. Useful information is shared indirectly instead of being wasted in an asocial learning environment.

We notice the efficiency of soft regulation is impacted by the following factors:

1. *Population*: Because of noise, recommendation is subject to uncertainty. However, when *n* ≫ 1, the variance becomes negligible, and the recommendation becomes deterministic (very close to mean) and accurate. This dependence on population size is intuitive: The information aggregated from a large population should be more useful compared to the one from a small population.
2. *Process*: We have proved that soft regulation with crowd recommendation preserves optimality. The advantage of the mechanism, however, is especially pronounced when the system is very noisy and the payoff function is very sensitive. A rule of thumb for the agents would be when a large sensitivity of the process is observed (either because of high noise or large curvature), the agents may be better off relying on the recommendation. Uncertainty drives the system towards cooperation. Soft regulation can potentially stabilize an open loop unstable process. This result also provides some insights on the wisdom of crowds. For example, the average performance can outperform the best individual when the system is very uncertain. In that sense, the “expert” knowledge may not be as useful in an emerging industry as the collective wisdom.
3. *Agent confidence level*: From the mathematical proofs and simulation evidence, we conclude that the best confidence level should be large but not too close to 100%. This is especially true when the system is very noisy and the process is very sensitive. In such setting, agents should put a substantial amount of trust on the regulator’s recommendations. Because of the trade-off between consensus and efficiency, in the early stage of soft regulation, the confidence level should be kept low for recommendation to quickly converge. As time proceeds, agents can be more and more confident regarding the recommendation.

Despite the name, soft regulation has applications beyond industrial regulation. The soft regulator module, i.e., $x_{i}^{+}=\left(1-\beta_{i}\right){\bar{x}}_{i}+\beta_{i}u$, can be integrated in different control systems and problem-solving scenarios (see Table 3). We only analyze a specific and stylized model in this paper to illustrate the efficacy of the mechanism. In practice, soft regulation should be implemented and modified in a case by case manner. For example, when the regulator can obtain more information other than the average action, it is entirely reasonable to formulate a better recommendation based on the richer information set such as trends, histograms, etc. The agents, instead of adjusting confidence level via the method proposed in this paper, can also explore and compare utilities (on a much slower timescale) to adapt new confidence levels. For a large population where centralized information collection is impractical, soft regulation might be plausible on a peer-to-peer basis. All these possibilities will be explored and analyzed in future work.

**Table 3:** Soft regulation applications.

Action	→	Utility
**health behavior**: e.g., sleep habit, exercise frequency, diet, etc.	→	**health condition** e.g., sleep quality, BMI, etc.
**operating condition**: e.g., *T*, *P*, feed ratio, flow rate, catalyst, etc.	→	**yield** e.g., production rate, etc.
**workplace environment**: e.g., indoor temperature, lighting, etc.	→	**productivity** e.g., profitability, etc.
**infrastructure planning**: e.g., traffic light control, hospital resource, budget allocation, etc.	→	**efficiency** e.g., congestion time, etc.

The medical domain is another applicable area of soft regulation. Powered by mobile phones and wearables, researchers can now collect timely mass medical data (via Apple’s ResearchKit [32] for example). Soft regulation is suitable in this scenario because medical research satisfies all three features, i.e., imperfect information (unknown relationships between patient behaviors and health conditions), weak interaction (one patient’s condition is not affected by another’s), and bounded rationality (patients always wish to improve their own health, however, have limited information). In addition, thanks to the convenience of mobile devices, we expect good participation rate. A large population size further ensures the accuracy of recommendation. Patients can optimize their own health while contributing to medical research. Even if patients do not want to optimize themselves, medical researchers may implement the soft regulation module to do that based on data collected locally. The confidence level can also be explicitly controlled by the service provider. Soft regulation in this setting becomes a crowdsourcing framework. The results in this paper are expected to hold.

This work also has some implications other than our central arguments on control and regulation. It reinforces the idea that an averaged opinion can accurately predict under uncertainty, i.e., the wisdom of crowds, given the population is large, independent, and relevant. Unlike conventional takes on the wisdom of crowds, soft regulation does not stop at collecting average information but also feeds it back to the system. This dynamical mechanism suggests more flexible scenarios and applications.

## Supporting Information

S1 Appendix Proofs and detailed derivations. In the Appendix, we show that soft regulation with crowd recommendation is optimal and robust when subjected to bounded noises when 0 ≤ *β*_*i*_ < 100%. We also discuss how confidence level affects the efficiency of this mechanism. In addition, we formulate a closed-form analytical solution for the simulation case study.
(PDF)
