# Supplementary material for: Soft Regulation with Crowd Recommendation: Coordinating Self-Interested Agents in Sociotechnical Systems under Imperfect Information
Source: PLoS One. 2016 Mar 15;11(3):e0150343. doi: 10.1371/journal.pone.0150343 (PMC4792447; doi:10.1371/journal.pone.0150343)
Supplement: S1 Appendix — In the Appendix, we show that soft regulation with crowd recommendation is optimal and robust when subjected to bounded noises when 0 ≤ βi < 100%. We also discuss how confidence level affects the efficiency of this mechanism. In addition, we formulate a closed-form analytical solution for the simulation case study. (PDF) [file pone.0150343.s001.pdf]

Soft Regulation with Crowd Recommendation: Coordinating Self-Interested Agents in Sociotechnical Systems under Imperfect Information

Supporting Information
S1 Appendix

Yu Luo, Garud Iyengar, Venkat Venkatasubramanian

Last updated: March 4, 2016 (version 23)

Optimality and robustness

The i-th agent follows the soft regulation dynamics $x_i^+ = h_i(x_i) = (1 - \beta_i)g_i(x_i) + \beta_i \sum_j x_j/n$. Let $\mathbf{x} \equiv [x_1, \ldots, x_n]^T \in \mathbb{X}^n$ denote the state vector of an n-agent system where action x_i of the i-th agent is restricted to belong to a bounded set $\mathbb{X} \subseteq \mathbb{R}$. The system-wide nominal (no noise) soft regulation map of the n-agent system is $\mathbf{x}^+ = H(\mathbf{x})$. Suppose the system is open loop optimal (i.e., every agent's action converges to the optimal action eventually). The optimal action $\theta^* = \arg\max_{x \in \mathbb{X}} f_i(x)$ should be the unique and attracting fixed point of each g_i map.

We first show that soft regulation with crowd recommendation does not alter the contraction property of the open loop process if $0 \leq \beta < 1$. Therefore, if the open loop system is optimal, so does soft regulation. We then show that the optimal state is stable and robust when subjected to bounded disturbances because there exists a continuous and smooth Lyapunov function for H.

Assumption 1. *The function g_i is differentiable for all $1 \leq i \leq n$. Furthermore, $|g_i'(x)| < 1$ for all $1 \leq i \leq n$ and $x \in \mathbb{X}$.*

Theorem 1. *The spectral radius $\rho(J(\mathbf{x}))$ of the Jacobian matrix of the soft regulation map H at $\mathbf{x}$ is at most $m = \max_{1 \leq i \leq n, x \in \mathbb{X}} \Big[(1 - \beta_i)|g_i'(x)| + \beta_i\Big]$.*

Proof. The Jacobian matrix of H is

$$J(\mathbf{x}) = \begin{bmatrix} (1-\beta_1)g_1'(x_1) & \dots & 0 \\ \vdots & \ddots & \vdots \\ 0 & \dots & (1-\beta_n)g_n'(x_n) \end{bmatrix} + \frac{1}{n}\begin{bmatrix} \beta_1 & \dots & \beta_n \\ \vdots & \ddots & \vdots \\ \beta_1 & \dots & \beta_n \end{bmatrix}$$

i.e., $J = D + \mathbf{u}\mathbf{b}^T$ where $D = \text{diag}((1-\beta_1)g_1', \dots, (1-\beta_n)g_n')$, $\mathbf{u} = [1, \dots, 1]^T$, and $\mathbf{b} = [\beta_1/n, \dots, \beta_n/n]^T$. Note that we drop x_i from $g_i'(x_i)$ for simplicity. The induced 1-norm $\|J(\mathbf{x})\|_1$ of matrix J is given by

$$\|J(\mathbf{x})\|_1 = \max_{\|\mathbf{v}\|_1=1} \|J(\mathbf{x})\mathbf{v}\|_1 = \max_{1\le i\le n} \Big[(1-\beta_i)|g_i'(x_i)| + \beta_i\Big] = m.$$

The result follows from noting that $\rho(J(\mathbf{x})) \le \|J(\mathbf{x})\|_1 = m$. □

This proof implies under Assumption 1 the soft regulation map H also contracts. Soft regulation with crowd recommendation preserves the contraction property.

Theorem 2. *The fixed point of the nominal soft regulation map H is asymptotically stable if $0 \le \beta_i < 1$.*

Proof. Since $m < 1$ for all $0 \le \beta_i < 1$. The fixed point $\mathbf{x}^* = [\theta^*, \dots, \theta^*]^T$ of H is asymptotically stable because $\rho(J(\mathbf{x})) \le m < 1$. □

This proof is also applicable for time-varying g_i. As long as $g_i(x,t)$ contracts for $t > T$ and $T < \infty$, the optimality result will hold. In reality, the optimization map g_i is subjected to noise because of the noisy utility f_i.

Theorem 3. *The fixed point $\mathbf{x}^*$ of H is robust when subjected to bounded disturbances.*

Proof. Let $V(\mathbf{x}) = \|\mathbf{x}-\mathbf{x}^*\|_1$, i.e., the Manhattan distance or ℓ_1-norm between the current state $\mathbf{x}$ and the optimal state $\mathbf{x}^*$. Since $0 \le m < 1$, we have that $(1-m)\|\mathbf{x}-\mathbf{x}^*\|_1 \le V(\mathbf{x}) \le (1+m)\|\mathbf{x}-\mathbf{x}^*\|_1$. According to mean value theorem for vector-valued function, $\|H(\mathbf{x}) - H(\mathbf{x}^*)\|_1$ is bounded by $m\|\mathbf{x}-\mathbf{x}^*\|_1$ where m is the matrix norm upper bound of the Jacobian for H. Therefore, we have

$$\begin{aligned} V(H(\mathbf{x})) - V(\mathbf{x}) &= \|H(\mathbf{x}) - \mathbf{x}^*\|_1 - \|\mathbf{x}-\mathbf{x}^*\|_1 \\ &= \|H(\mathbf{x}) - H(\mathbf{x}^*)\|_1 - \|\mathbf{x}-\mathbf{x}^*\|_1 \\ &\le (m-1)\|\mathbf{x}-\mathbf{x}^*\|_1. \end{aligned}$$

Thus, the continuous function $V(\mathbf{x})$ is a suitable Lyapunov function for H. According to stability theory [1], the optimal state is robust when subjected to bounded disturbances and can be restored. □

Efficiency

We measure the performance of soft regulation using mean squared error (MSE) defined as follows:

$$\text{MSE} = \frac{1}{n}\sum_i (x_i - \theta^*)^2 = \frac{1}{n}\|\mathbf{x} - \mathbf{x}^*\|_2^2.$$

Theorem 4. *If $\beta_i \equiv \beta$, the largest singular value of the Jacobian $J(\mathbf{x})$ of H is bounded by $m = (1-\beta)g_m + \beta$ where $g_m = \max_i |g_i'|$.*

Proof. $\|J(\mathbf{x})\|_2$ denotes the largest singular value (square root of the largest eigenvalue of $J^T J$). For $\beta_i = \beta$, the Jacobian is reduced to

$$\begin{aligned} J_{ij} &= \frac{\partial h_i}{\partial x_j} \\ &= \begin{cases} (1-\beta)g_i' + \beta/n & (j = i) \\ \beta/n & (j \neq i) \end{cases}. \end{aligned}$$

In vector notation, $J = (1-\beta)G + \beta \mathbf{a}\mathbf{a}^T$ where $G = \operatorname{diag}(g_1', \ldots, g_n')$ and $\mathbf{a} = \mathbf{1}/\sqrt{n}$ such that $\|\mathbf{a}\|_2 = 1$. To determine the largest singular value, we solve the following optimization problem:

$$\begin{aligned} \max_{\|\mathbf{v}\|_2=1} \mathbf{v}^T J^T J \mathbf{v} &= \max_{\|\mathbf{v}\|_2=1} \left\{ (1-\beta)^2 \mathbf{v}^T G^T G \mathbf{v} + 2\beta(1-\beta)\left[(G^T\mathbf{a})^T\mathbf{v}\right]^T (\mathbf{a}^T\mathbf{v}) + \beta^2 \mathbf{v}^T \mathbf{a}\mathbf{a}^T \mathbf{v} \right\} \\ &\leq (1-\beta)^2 \max_i g_i^2 + \beta^2 + 2\beta(1-\beta) \max_{\|\mathbf{v}\|_2=1} \left|(G^T\mathbf{a})^T\mathbf{v}\right| \cdot \left|\mathbf{a}^T\mathbf{v}\right| \\ &\leq (1-\beta)^2 \max_i g_i^2 + \beta^2 + 2\beta(1-\beta)\left|(G^T\mathbf{a})^T\right| \\ &\leq (1-\beta)^2 \max_i g_i^2 + \beta^2 + 2\beta(1-\beta) \max_i |g_i'| \\ &= \left[(1-\beta)\max_i |g_i'| + \beta\right]^2 \\ &= m^2. \end{aligned}$$

Therefore, the largest singular value of J is bounded by m. □

We introduce i.i.d. noise ω_i such that $\mathbb{E}[\omega_i] = 0$, $\mathbb{E}[\omega_i^2] = \sigma_\omega^2$, and noise vector $\mathbf{w} = [\omega_1, \ldots, \omega_n]^T$. The noisy exploration algorithm is therefore $g_i(x_i) + \omega_i$. Here we are interested in how β affects the MSE. In order to do so, we simplify the system such that $\beta_i = \beta$. According to mean value theorem and the theorem above, we have $\|H(\mathbf{x}) - H(\mathbf{x}^*)\|_2 \leq m\|\mathbf{x} - \mathbf{x}^*\|_2$. Therefore, the MSE progression can be computed as follows:

$$\begin{aligned}
\mathbb{E}[\text{MSE}^+] &= \frac{1}{n}\mathbb{E}\Big[\|\mathbf{x}^+ - \mathbf{x}^*\|_2^2\Big] \\
&= \frac{1}{n}\mathbb{E}\Big[\|H(\mathbf{x}) - H(\mathbf{x}^*) + (1-\beta)\mathbf{w}\|_2^2\Big] \\
&= \frac{1}{n}\|H(\mathbf{x}) - H(\mathbf{x}^*)\|_2^2 + \frac{(1-\beta)^2}{n}\mathbb{E}\Big[\|\mathbf{w}\|_2^2\Big] \\
&\leq \frac{m^2}{n}\|\mathbf{x} - \mathbf{x}^*\|_2^2 + (1-\beta)^2\sigma_\omega^2 \\
&= m^2\,\text{MSE} + (1-\beta)^2\sigma_\omega^2
\end{aligned}$$

where $m = (1-\beta)g_m + \beta$. The steady-state MSE is bounded by

$$\begin{aligned}
\lim_{t\to\infty}\mathbb{E}[\text{MSE}(t)] &\leq \frac{(1-\beta)^2\sigma_\omega^2}{1-m^2} \\
&= \frac{1-\beta}{1+m}\frac{\sigma_\omega^2}{1-g_m} \\
&= \frac{1-m}{1+m}\frac{\sigma_\omega^2}{(1-g_m)^2}.
\end{aligned}$$

Note that as β increases (m increases), the steady state error $\lim_{t\to\infty}\mathbb{E}[\text{MSE}(t)]$ decreases. Let $\Delta\,\text{MSE} = \text{MSE} - (1-m)\sigma_\omega^2/[(1+m)(1-g_m)^2]$ denote the deviation from the steady-state error. We have

$$\mathbb{E}[\Delta\,\text{MSE}^+] \leq m^2 \Delta\,\text{MSE}\,.$$

As β increases, the speed of convergence decreases as well. There is a trade-off between accuracy and speed. This further implies that at a finite time t, there should exist an optimal β^* between 0 and 1 such that the MSE is the smallest.

Closed-form solution

Recall Kiefer-Wolfowitz [2] stochastic gradient method

$$g(x) = x + a_t\frac{f(x+c_t) - f(x-c_t)}{c_t}. \tag{1}$$

The updated state x_i^+ implied by this gradient-based update scheme is given by

$$x_i^+ = (1-\beta)(1-4ka_t)x_i + \beta u + a_t \cdot \frac{1-\beta}{c_t} \cdot \hat{\omega}_i \tag{2}$$

where $\hat{\omega}_i \sim \mathcal{N}(0, \sqrt{2}\sigma_\omega)$ is the effective noise resulting from computing the discrete approximation to the gradient in (1). Recall that the crowd recommendation is $u = \sum_i x_i/n$. Thus, the updated u^+ of the recommendation is given by

$$u^+ = \Big[1 - 4ka_t(1-\beta)\Big]u + a_t \cdot \frac{1-\beta}{c_t} \cdot \frac{1}{n}\sum_i \hat{\omega}_i.$$

Let $U_t = \mathbb{E}[u(t)]$. Since $\mathbb{E}[\hat{\omega}] = 0$, we have that

$$U_{t+1} - U_t = -4ka_t(1-\beta)U_t.$$

Note that the variance of $u(t+1)$ is $2a_t^2(1-\beta)^2\sigma_\omega^2/(c_t^2 n) \ll 1$ for $n \gg 1$. Therefore, compared to $x_i(t)$, the recommendation $u(t)$ can be safely treated as a deterministic variable i.e., $u(t) \approx U_t$. Especially when $|4ka_t(1-\beta)| \ll 1$, one can approximate the difference equation by the ODE

$$\frac{\mathrm{d}U_t}{\mathrm{d}t} = -4ka_t(1-\beta)U_t.$$

Therefore,

$$U_t \approx U_{t_0} \exp\left[-4k(1-\beta)\int_{t_0}^{t} a_\tau \mathrm{d}\tau\right].$$

Thus, we expect U_t to quickly converge to θ^* (or 0 in this case) unless β is too close to 1. In addition, as k increases, U_t approaches θ^* faster.

From (2) it follows that

$$\begin{aligned}
\mathrm{MSE}_{t+1} \equiv& \frac{1}{n}\sum_i x_i^2(t+1) \\
=& (1-\beta)^2(1-4ka_t)^2\frac{1}{n}\sum_i x_i^2(t) + \beta^2 u(t)^2 \\
&+ 2(1-\beta)(1-4ka_t)u(t)\frac{1}{n}\sum_i x_i(t) + \frac{1}{n}a_t^2\frac{(1-\beta)^2}{c_t^2}\sum_i \hat{\omega}_i^2 \\
&+ \frac{1}{n}\sum_i \Big[(1-\beta)(1-4ka_t)x_i(t) + \beta u(t)\Big]a_t\frac{(1-\beta)}{c_t}\hat{\omega}_i.
\end{aligned}$$

Since $u \equiv \sum_i x_i/n$, $u(t) \approx U_t$, and $\mathbb{E}[\hat{\omega}_i] = 0$, we have

$$\mathbb{E}\left[\mathrm{MSE}_{t+1}\right] = (1-\beta)^2(1-4ka_t)^2\mathbb{E}\left[\frac{1}{n}\sum_i x_i^2(t)\right] + \beta\Big[2(1-\beta)(1-4ka_t)+1\Big]U_t^2 + 2a_t^2\frac{(1-\beta)^2}{c_t^2}\sigma_\omega^2.$$

We therefore define $X_t \equiv \mathbb{E}[\mathrm{MSE}_t]$. It follows that the update for X_t is given by recursion

$$X_{t+1} = A_t X_t + B_t$$

where

$$A_t = (1-\beta)^2(1-4ka_t)^2$$

and

$$B_t = \beta\Big[2(1-\beta)(1-4ka_t)+1\Big]U_t^2 + 2a_t^2\frac{(1-\beta)^2}{c_t^2}\sigma_\omega^2.$$

Since $\lim_{t\to\infty} A_t = A_\infty$, $\lim_{t\to\infty} B_t = B_\infty$, and $\lim_{t\to\infty} X_{t+1} = B_\infty/(1-A_\infty)$, one would expect that when t is large,

$$\begin{aligned} X_{t+1} &\approx \frac{B_t}{1-A_t} \\ &= \frac{2a_t^2(1-\beta)^2\sigma_\omega^2/c_t^2 + \beta\Big[2(1-\beta)(1-4ka_t)+1\Big]U_t^2}{1-(1-\beta)^2(1-4ka_t)^2}. \end{aligned} \tag{3}$$

This approximation in fact agrees with the simulation (see lines in all simulation result figures).

From (3), it follows that X_{t+1} converges to 0. Meanwhile, when confidence level is low, i.e., $\beta \approx 0$ (explorers), the dependence on U_t vanishes very quickly, and (3) can be simplified as follows:

$$X_{t+1} \approx \frac{2a_t^2\sigma_\omega^2/c_t^2}{1/(1-\beta)^2 - (1-4ka_t)^2}$$

and X_{t+1} monotonically *decreases* as β increases. On the other hand, when confidence level is high, i.e., $\beta \approx 1$ (followers), U_t^2 term dominates σ_ω^2, one can simplify (3) to

$$X_{t+1} \approx \beta\left\{U_{t_0}\exp\left[-4k(1-\beta)\int_{t_0}^{t} a_\tau \mathrm{d}\tau\right]\right\}^2$$

and X_{t+1} monotonically *increases* as β increases.

References

[1] Teel AR. Discrete Time Receding Horizon Optimal Control: Is the Stability Robust? In: Optimal Control, Stabilization and Nonsmooth Analysis. Springer; 2004. p. 3–27.

[2] Kiefer J, Wolfowitz J, et al. Stochastic estimation of the maximum of a regression function. The Annals of Mathematical Statistics. 1952;23(3):462–466.
